# Metastatic breast cancer in resource-limited settings: insights from a retrospective cross-sectional study at a radiotherapy centre in Sub-Saharan Africa

**DOI:** 10.3332/ecancer.2025.1955

**Published:** 2025-07-29

**Authors:** Joseph Daniels, Letlhogonolo Ernity Mosadi, Andrew Yaw Nyantakyi, Edwina Ayaaba Ayabilah, Judith Naa Odey Tackie, Kofi Adesi Kyei

**Affiliations:** 1 National Centre for Radiotherapy, Oncology, and Nuclear Medicine Centre, Korle-Bu Teaching Hospital, Accra PO Box KB 369, Ghana; 2 Department of Radiography, University of Ghana, Accra PO Box KB 143, Ghana; 3 Department of Oncology, Cape Coast Teaching Hospital, Cape Coast PO Box CT 1363, Ghana

**Keywords:** molecular subtype, breast cancer, metastasis, TNBC, HER-2 positive

## Abstract

**Background:**

Metastatic breast cancer (MBC) is a significant cause of cancer-related mortality worldwide, with unique challenges in resource-limited settings. Radiotherapy centers play a critical role in the management of MBC, but there is limited data on the clinical and pathological profiles as well as treatment patterns in these settings.

**Aim:**

To describe the socio-demographic characteristics, clinico-pathological features, molecular profiles and treatment patterns of patients with MBC at a major radiotherapy centre in sub-Saharan Africa

**Methods:**

This was a quantitative retrospective cross-sectional study involving patients with MBC managed between 2016 and 2020. Data were extracted from patients’ medical records and analysed with STATA software (version 16). Descriptive statistics were used to summarise the data.

**Results:**

The study involved 154 MBC patients with a median age of 48 years (IQR 19) ranging from 26 to 79 years. Young adults (< 39 years) comprised 29.9% whereas patients ≥60 years accounted for 12.9%. Triple-negative breast cancers comprised 28.6% whereas human epidermal growth factor receptor-2/Neu – enriched tumours constituted 18.8%. Only 29.9% were diagnosed with de novo metastasis. A considerable majority (85.1%) were treated with palliative intent whereas 14.9% received best supportive care only, with none receiving curative treatment. The sites of first occurrence of distant metastasis were bone tissue (38.3%), lung (34.4%), liver (14.9%) and the brain (12.4%). Overall, 63% had solitary metastatic sites whereas 27.9% and 7.8% had double and triple metastatic sites, respectively. About a quarter (24.7%) presented to the radiotherapy center within 6 months of the onset of symptoms, whereas the majority (84.5%) sought care within 24 months.

**Conclusion:**

Bone, lung, liver and brain were the primary metastatic sites, with complex combinations involving these organs, reflecting the heterogeneity of the disease. Context-specific strategies are needed to address the high burden of advanced-stage disease and improve oncological care for patients with MBC in limited-resource settings.

## Introduction

Breast cancer remains the most common malignancy affecting women globally, with steadily rising incidence rates, expected to double by 2035 [1]. Breast cancer accounts for approximately 2.3 million new cancer diagnoses each year, representing about 11.7% of all cancer cases globally [2]. Advanced breast cancer (ABC) accounts for a high proportion of cancer-related deaths among women [3], with annual mortality of about 8.8 million people [1]. Breast cancer is the leading cause of cancer-related deaths among Ghanaian women who also tend to present for treatment at advanced stages (stages III and IV) [4, 5]. Metastasis-related complications account for more than 90% of fatalities among patients with breast cancer [6]. While early-stage breast cancer can often be treated effectively, metastatic breast cancer (MBC) remains a major public health challenge, particularly in limited-resource settings. In all, patients are often diagnosed with advanced disease due to late presentation and limited access to early detection and treatment services [7]. Sub-Saharan Africa exemplifies such settings, where the burden of breast cancer, including MBC, continues to grow, with dire consequences for both patients and healthcare systems. MBC, characterised by the spread of cancer to distant organs such as the bones, liver, lungs and brain, remains the most lethal form of breast cancer, significantly reducing survival rates [8]. In high-income countries, early detection programs and advanced therapeutic options have significantly improved breast cancer outcomes [9]. In these settings, only about 6%–10% of breast cancer patients present with metastatic disease at diagnosis [10]. In contrast, limited-resource settings, such as sub-Saharan Africa, face numerous challenges This results in poorer outcomes for breast cancer patients, with up to 50% of breast cancer patients presenting with metastatic disease [11]. A recent study in Ghana reported that the breasts were the second most frequent site of occurrence of de novo metastatic cancer (17.6%) among adult patients managed between 2021 and 2022 [12].

Breast cancer has a proclivity to metastasize to bone (50%–65%), lung (17%), brain (16%) and liver (6%) [13]. The symptoms of metastatic disease depend on the exact site and include headaches, seizures, bone pain, vomiting, pain in the chest or abdomen and dyspnea [14]. Breast cancer metastasis is influenced by tumour size, histologic grade and nodal involvement as well as hormone receptor status. Once metastasis occurs, the disease is considered incurable, with treatment options primarily focused on prolonging survival and alleviating symptoms [6]. This is in spite of advances in the treatment of MBC that have significantly improved patient survival. Even when breast cancer is detected early, there is still a 20%–30% chance of developing metastatic disease [14]. In limited-resource settings, the management of MBC is fraught with multiple challenges. Access to advanced diagnostic tools, such as imaging and biopsy, is often limited, making it difficult to accurately assess the extent of disease spread [15]. Most breast cancer fatalities are caused by metastasis to other organs rather than the primary tumour [14]. An estimated 5%–10% of patients have de novo metastasis at the time of diagnosis and following therapy, 20%–40% of early breast cancer patients experience recurrence and distant metastasis [16].

Therapeutic options are often constrained by the limited availability of systemic therapies, such as chemotherapy, targeted therapies and hormonal agents. Even when these treatments are available, their cost often places them beyond the reach of most patients [17]. Furthermore, many healthcare facilities in sub-Saharan Africa lack the infrastructure needed for the safe administration of these treatments, including oncology pharmacies and infusion centers. Palliative care, which is essential for managing symptoms in patients with metastatic disease, is also limited in many parts of the region [18].

Understanding the epidemiological patterns and clinicopathological profiles of patients with MBC in sub-Saharan Africa is essential for informing public health strategies, improving resource allocation and developing context-appropriate treatment protocols. The aim of the study was to assess the socio-demographic characteristics, clinico-pathological features, molecular profiles and treatment patterns of patients with MBC at a major oncology and radiotherapy centre in sub-Saharan Africa. By focusing on MBC in sub-Saharan Africa, the study contributes to the global fight against breast cancer to ensure that all patients, regardless of geographic location, have access to the care they need.

## Methods

### Study design and setting

The research was a quantitative retrospective cross-sectional study conducted at the largest radiotherapy and cancer treatment centre in Ghana which also serves as a major radiotherapy referral hub in the sub-region. The centre also provides systemic therapy (including chemotherapy, immunotherapy, targeted and hormonal therapy) and supportive care services to patients. The study period spanned from 01 January 2016 to 31 December 2020, capturing retrospective data from patient records to provide a snapshot of MBC management in a resource-limited healthcare environment.

### Participants

A total of 2,157 patients with confirmed breast cancer were managed between 2016 and 2020, out of which 863 had metastatic disease. A purposive sampling technique was used to select the case notes of eligible participants based on being adult female patients (≥ 18 years) with a histologically and radiologically confirmed diagnosis of MBC. Eligible patients recruited were either newly diagnosed with MBC or diagnosed with distant recurrence after prior treatment for breast cancer. Patients with metastatic lesions from other primary cancers were excluded. Eligible patients who did not have immunohistochemical reports on estrogen, progesterone and human epidermal growth factor receptor-2(HER-2/neu) status were also excluded. Similarly, patients with locoregional breast cancer recurrence without distant metastasis were also excluded from the study even if they were managed with palliative intent or best supportive care only.

### Variables

- **Time intervals related to metastasis -** key time-related variables included the *‘onset-to-first visit’* interval, which captured the time between the appearance of initial symptoms and the patient’s first visit to the radiotherapy and oncology centre, reflecting potential delays in seeking and accessing the appropriate specialised cancer care. The *‘onset-to-metastasis’* interval measured the time from the initial onset of symptoms to the first confirmed diagnosis of distant metastasis, highlighting the timeline of disease progression. Additionally, the *‘first visit-to-metastasis’* interval tracked the duration between the patient’s initial visit to the radiotherapy centre and the diagnosis of distant metastasis, providing insights into potential diagnostic delays or disease progression after initial contact with the oncology healthcare system. These intervals were critical for evaluating care timelines, potential treatment delays and disease evolution in a resource-limited setting.
- **Molecular subtypes -** The determination and classification of the molecular subtypes of breast cancer, were undertaken using immunohistochemistry to assess key biomarkers, including estrogen receptor (ER), progesterone receptor (PR), HER-2/neu status and Ki-67proliferation index. In cases where immunohistochemistry results for HER2 were equivocal, fluorescence *in situ* hybridisation was employed to confirm HER2 status. Based on the expression of these biomarkers, invasive breast tumours were categorised into four primary molecular subtypes: luminal A, luminal B (HER-2/Neu+ and HER-2/Neu-), HER-2/Neu-enriched and triple negative breast cancer (TNBC).
- **Performance status -** The Eastern Cooperative Oncology Group (ECOG) scale was used to summarise the functional status of the cancer patients based on their ability to care for themselves, undertake physical activity and activities of daily living [19, 20].

### Data collection

The study utilised data extracted from patients’ hospital-based medical records and the institutional cancer registry. Key demographic information recorded included age, gender, marital status, highest level of education, residence and employment status. Clinical and tumour-related data included time to metastasis (de novo metastatic versus recurrent), ECOG performance status (0–4), breast cancer laterality, treatment intention, first site of metastasis as well as the total number and site(s) of distant metastasis. Pathological data included tumour grade, histology and molecular subtype as well as the expression of specific immunohistology biomarkers, namely: ER, PR, HER-2/neu and Ki-67 proliferative index.

### Bias

The use of purposive sampling ensured that only eligible patients were recruited in the study. However, selection bias may have arisen due to the inclusion of only patients who received treatment at the oncology and radiotherapy centre, possibly excluding those with metastatic disease who were either not referred for oncological care or those who sought alternative treatments. Patients’ history and clinical data could not be independently verified due to the reliance on retrospective medical records. However, pathological, radiological and treatment-related information were independently verified by reviewing original images, reports and test results. Data extraction procedures were standardised with strict and uniform application of the eligibility criteria.

### Data management and analysis

Data management procedures included an independent review of patients’ records to ensure the accuracy of extracted data. Eligible patients with incomplete or missing clinical and/or pathological records were excluded from the analysis to maintain the integrity of the dataset. The quantitative data collected were initially recorded in a Microsoft Excel worksheet and analysed with STATA version 17. Descriptive statistics, including mean and standard deviation were calculated for continuous variables, while frequency and percentages were determined for categorical variables. Data were graphically presented as graphs and frequency tables.

### Ethical considerations

Ethical approval was as obtained from the Ethical and Protocol Review Committee of the School of Biomedical and Allied Health Sciences, University of Ghana, Legon prior to the commencement of the study. Patients’ data were discreetly managed with utmost confidentiality. All patient-identifying information were removed prior to data analysis. Written informed consent was obtained from all patients either directly or through a legally authorised representative.

## Results

### Socio-demographic and clinical characteristics

**Figure 1.**
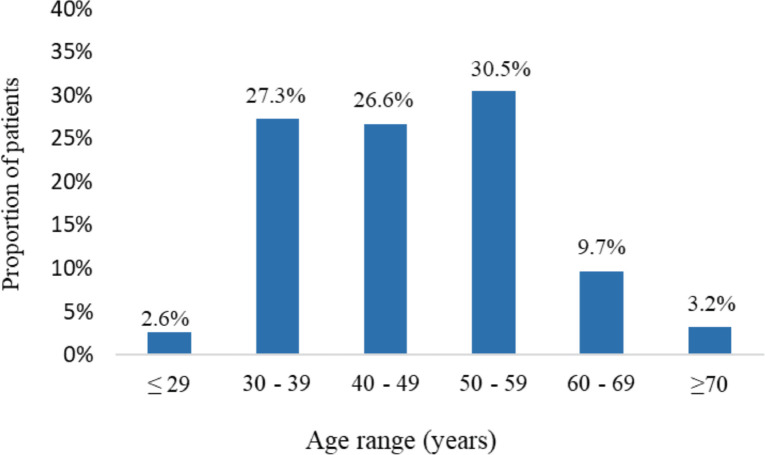
Age distribution of participants (*N* = 154).

The study involved 154 patients with a confirmed diagnosis of MBC. The median age was 48 years (IQR 19) ranging from 26 to 79 years. In all, 2.6% were younger than 29 years whereas 3.2% were ≥70 years. Young adults (patients ≤39 years) comprised 29.9% whereas patients who were ≥60 years accounted for 12.9% (Figure 1).

A considerable majority (61%) was employed whereas 27.9% were unemployed (Table 1). In all, 14.9% attained tertiary education whereas 12.4% were not formally educated. Most (68.8%) were urban dwellers whereas 31.2% resided in rural areas. Premenopausal women comprised 57.1% whereas postmenopausal women accounted for 42.9%. Some of the participants had a good performance status of ECOG 0 (12.3%) or ECOG 1 (15.6%) whereas close to half (47.4%) were classified as ECOG 2. Also, 20.1% were ECOG 3 whereas 4.6% were moribund with ECOG 4 performance status.

### Tumour- and treatment-related characteristics

In all, 51.3% had left-sided tumours whereas 46.1% and 2.6% had right-sided and bilateral breast cancer, respectively. The predominant histological type was invasive carcinoma, NST (91.6%). Also, 46.7% had grade III tumours whereas 15.6% had grade I tumours. TNBCs comprised 28.6% whereas HER-2/Neu – enriched tumours constituted 18.8%. Also, 22.1% were of the luminal A molecular subtype. Approximately 50% had undergone mastectomy prior to the diagnosis of distant metastasis whereas 22.1% had undergone breast-conserving surgery (Table 2). In all, only 29.9% were diagnosed with de novo metastasis. A considerable majority (85.1%) were treated with palliative intent whereas 14.9% received best supportive care only, with none receiving curative treatment. The most frequent sites of first metastasis were bone tissue (38.3%) and lung (34.4%). Overall, 63% had solitary metastatic lesions whereas 27.9% and 7.8% had double and triple metastatic sites, respectively.

**Figure 2.**
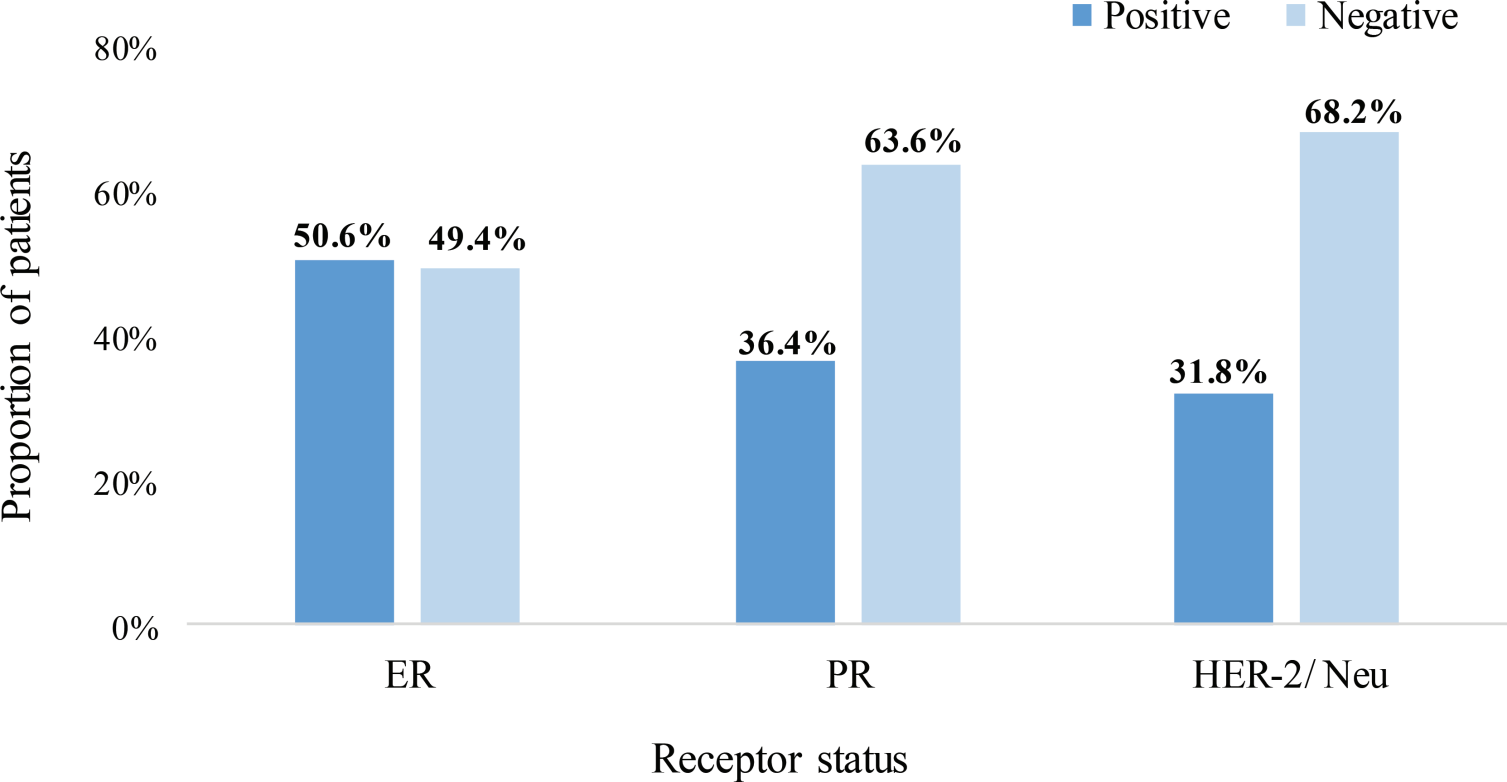
Hormone and HER-2/neu receptor status. ER: estrogen receptor, PR: progesterone receptor, HER-2/Neu: human epidermal growth factor receptor – 2.

Approximately equal proportions of patients had positive (50.6%) and negative (49.4%) ER status. Also, 36.4% were PR – positive whereas only 31.8% were HER-2/Neu+ as illustrated in Figure 2.

**Figure 3.**
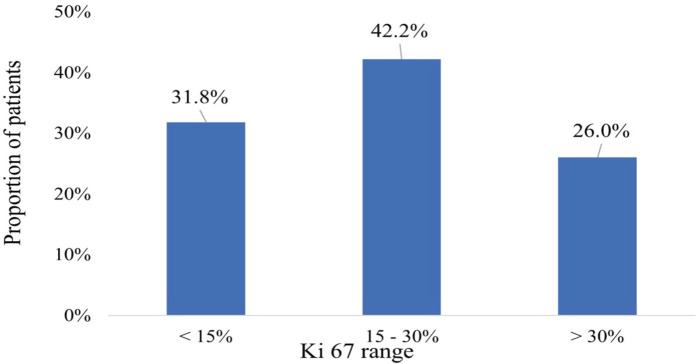
Ki-67 proliferative index.

The Ki-67 proliferative index scores of the participants ranged from 5% to 70%. A considerable majority (42.2%) had scores between 15% and 30% whereas 31.8% had scores ≤15%. Additionally, 26% had scores >30% as shown in Figure 3.

**Figure 4.**
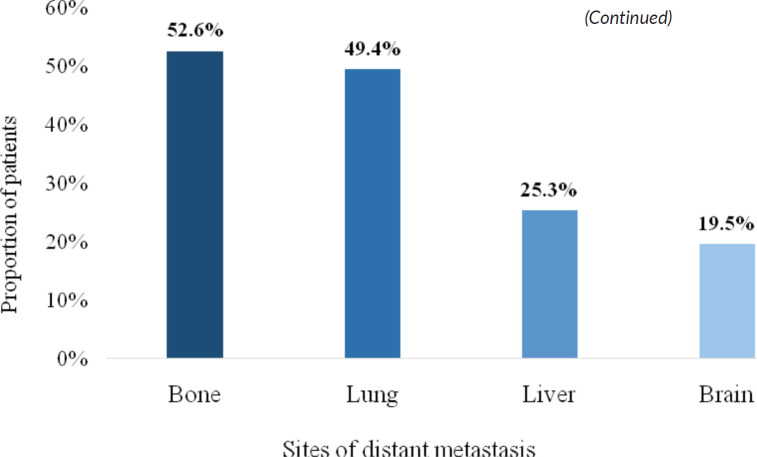
Sites of distant metastasis.

There were four different sites of occurrence of distant metastasis. The most frequent site of distant metastatic spread was bone (52.6%), followed by the lung (49.4%), liver (25.3%) and brain (19.5%) (Figure 4).

Table 3 provides a summary of the patterns and sequence of occurrence of distant metastasis, reflecting a complex landscape of metastatic spread that varies widely in both the initial site and combination of sites of occurrence of metastasis. Among patients with solitary metastasis, a considerable proportion experienced bone-only metastasis (39.1%). The lung was the second most frequent site for isolated metastases (33%), followed by the liver (16.5%) and brain (11.4%). Regarding patients with double metastatic sites (*n* = 43), the most frequent first sites of metastasis were bone (39.5%) and lung (37.3%) whereas the most frequent second sites of distant spread were the lungs (32.5%), followed by bone (30.2%) and liver (23.3%). Combinations involving lung and bone were notable, with the pairing accounting for 44.2% of all cases of dual-distant metastatic spread. Additionally, concurrent lung and liver metastases occurred in 20.9% of the patients with dual metastasis. While most patients had either solitary or dual-organ metastases, some presented with more complex patterns involving three or more organs. For example, concurrent lung, liver and bone metastases were observed in 50% of those with triple metastatic sites (*n* = 12) whereas 8.3% experienced concurrent lung, liver and brain metastasis.

**Figure 5.**
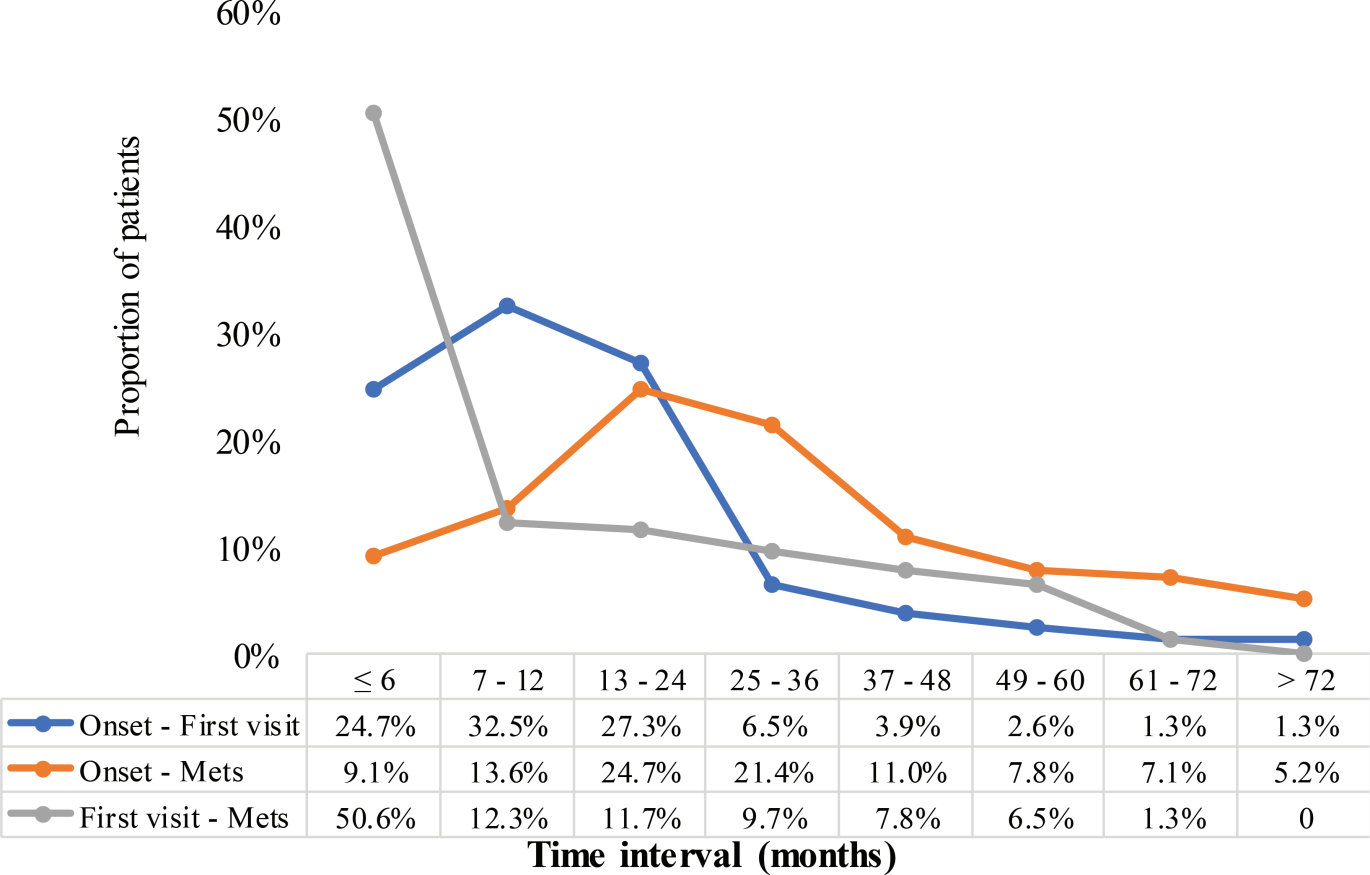
Duration of development of distant metastasis. Mets: distant metastasis. Blue line: ‘Onset – First visit’ – represents the time interval between the appearance of initial symptoms and patients’ first visit to the radiotherapy and oncology centre. Orange line: ‘Onset – Mets’ – represents the time interval between the initial onset of symptoms and the first confirmed diagnosis of distant metastasis. Grey line: ‘First visit – Mets’ – indicates the time interval between patients’ initial visit to the radiotherapy and oncology centre and the diagnosis of distant metastasis.

Figure 5 illustrates the distribution of MBC patients across different time spectra regarding three key time-related intervals: onset – first visit, onset – metastasis and first visit – metastasis. The data reveal patterns of how quickly patients sought care after the onset of symptoms, progression to distant metastasis and the elapsed time between initial diagnosis and metastatic spread. About a quarter (24.7%) presented to the radiotherapy center within 6 months of the onset of symptoms, whereas the majority (84.5%) sought care within 24 months. However, 15.5% delayed visiting the center for more than 2 years, with a small subset (1.3%) reporting after >6 years. Only 9.1% of the patients developed metastases within the first 6 months, indicating that metastases often develop over longer periods. The highest proportion (24.7%) were diagnosed with metastasis within 13–24 months after symptoms-onset, however, 28.7% had metastatic disease diagnosed ≥3 years after symptoms-onset. A considerable proportion (50.6%) were diagnosed with distant metastasis within 6 months of their first visit. However, fewer patients (12.3%) developed metastases between 7 and 12 months, with declining proportions over longer periods. Notably, 5.2% of patients developed metastases more than 6 years after their first visit.

## Discussion

The retrospective analysis of patients with MBC managed at a leading radiotherapy centre in Ghana provides vital insights into the socio-demographic, clinical, molecular and therapeutic patterns of MBC in sub-Saharan Africa (SSA), emphasising key challenges typical in resource-constrained healthcare environments. While some sociodemographic trends such as the predominance of urban dwellers (68.8%) and relatively younger median age (48 years), align with previously reported patterns in SSA, the study goes further by offering granular data on tumour biology, metastatic patterns, treatment intent and timing of care-seeking behaviours. These findings have important clinical and policy implications, especially in the context of Ghana’s oncology-related healthcare system and ongoing global breast cancer initiatives (GBCIs).

The relatively low median age and the finding of 29.9% of patients being ≤39 years represents a pattern of early onset of breast cancer in African women, associated with more aggressive, high-grade tumours [21, 22]. Given the absence of a national screening program in Ghana, these patients often miss early detection opportunities. It may be prudent for individual cancer centers to adopt context-specific screening guidelines, especially targeting high-risk younger populations. Incorporating age-specific and risk-based recommendations that are tailored to local epidemiological data, into the national cancer control strategy could substantially improve early detection rates. The newly released ABC6 Consensus Guidelines by the ABC Global Alliance highlight the importance of personalised and equitable care in metastatic disease, including timely diagnosis, access to multidisciplinary care and support for younger patients [23]. These guidelines recommend patient navigation systems and psychosocial support for young women with MBC – services that are often lacking in Ghana. Ghanaian oncology institutions, supported by the Ministry of Health (MoH), could adopt select ABC6 recommendations, contextualising them within the available resources and health infrastructure.

The employment status and educational attainment data highlight socio-economic vulnerabilities that may influence health literacy, care-seeking behaviour, access to healthcare services and adherence to cancer treatment [24]. Public education campaigns on breast cancer, tailored in local languages and disseminated via community networks, radio and religious organisations, could help address the persistent knowledge and awareness gaps [25]. Ghana could draw lessons from its successful HIV/AIDS awareness campaigns, which combined health system reform with grassroots advocacy and international funding support [26]. A similar multi-sectoral approach for breast cancer awareness – integrated into the existing Community-based Health Planning and Services (CHPSs) system – could extend a reach to rural and marginalised populations.

A considerable proportion of patients had TNBC (28.6%), followed by luminal A (22.1%), HER2-enriched (18.8%), luminal B HER-2/neu- (17.5%) and luminal B HER-2/neu+ (13%) breast cancer. The high prevalence of TNBC and HER2-enriched subtypes underscores the aggressive disease profile of African women with breast cancer [27]. Triple-negative breast cancer is associated with poorer outcomes and limited treatment options, particularly in settings lacking targeted therapies [28]. Strengthening diagnostic capacity through investment in immunohistochemistry, in-country molecular profiling and centralised pathology services should be a priority for Ghana’s National Cancer Control Programme (NCCP). These investments are especially urgent given the rising burden of non-communicable diseases in the country. Ghana’s NCCP (2012–2016) made strides in establishing treatment centers but did not explicitly address MBC or advanced-stage treatment pathways [29]. An updated national cancer control strategy should explicitly include MBC care and identify roles for tertiary healthcare centers, teaching hospitals and international partners in expanding infrastructure. The MoH could also explore public-private partnerships and regional collaborations to improve access to imaging modalities like MRI and PET-CT, drawing lessons from countries like Egypt, which despite shared resource constraints, has made significant strides in imaging and cancer diagnostics [30].

Management approach was largely palliative (85.1%), with no patients receiving curative treatment. The palliative focus reflects limitations in healthcare infrastructure, access to early diagnostic services and availability of advanced treatments in SSA. In limited-resource settings, financial constraints, inadequate healthcare coverage and lack of specialised oncology services can prevent the widespread adoption of curative treatment strategies. These constraints highlight the need for adaptable treatment guidelines that prioritise the quality of life, especially given the high prevalence of advanced disease at diagnosis. The predominant use of the Cobalt-60 teletherapy machine (88%) highlights the reliance on older technologies, which are still the backbone of radiotherapy in many low-resource settings. The linear accelerator was used for 12% of the patients, underscoring limited access to more advanced equipment. Efforts to introduce linac-based treatments have been hampered by maintenance costs and power supply challenges in sub-Saharan Africa [31].

The metastatic patterns observed, with bone (52.6%), lung (49.4%) and liver (10.3%) as the most common sites of spread, emphasises the need for enhanced diagnostic capabilities. Bone-only metastases often cause debilitating complications, such as fractures and pain, and can significantly affect quality of life. The relatively low prevalence of brain-only metastases aligns with reports from limited-resource settings where advanced imaging tools for the early detection of brain metastasis are not widely accessible [31, 32]. Currently, access to bone scans and chest CT imaging is limited in many parts of Ghana, potentially leading to underdiagnosis of metastatic burden. Advocating for improved imaging infrastructure, particularly CT, MRI and bone scintigraphy, should be a central demand of clinicians and patient advocates. Given that nearly half of the patients were diagnosed with metastasis within 6 months of their first visit, this raises concerns about diagnostic delays and missed opportunities for earlier intervention.

The involvement of multiple metastatic sites (63%) complicates treatment for MBC, since such patients may require more comprehensive systemic therapies such as chemotherapy or targeted therapies that address the widespread nature of their disease. Combinations involving lung and bone were notable, with the pair accounting for 5.2% of cases. This sequence suggests a predilection for breast cancer cells to seed across both the pulmonary and skeletal systems, consistent with hematogenous dissemination pathways [33]. While most patients had either isolated or dual-organ metastases, some presented with more complex patterns involving three or more organs. For example, lung + liver + bone metastases were observed in a small fraction (1.3%). This complex spread pattern is rare but suggests a highly aggressive cancer phenotype, likely linked to poor outcomes. Interestingly, lung + bone + brain metastases were found in 0.7% of patients, reflecting the unpredictable nature of metastatic spread in advanced disease. The presence of bone involvement in nearly every multi-organ metastasis scenario further reinforces its role as a frequent target of breast cancer dissemination. This distribution of metastatic patterns has important implications for treatment strategies. In resource-limited settings, managing such diverse and advanced disease presentations poses a significant challenge, especially when access to specialised care, advanced imaging and targeted therapies is restricted. The management of bone metastases typically involves bisphosphonates or denosumab, while brain metastases may require radiotherapy, surgery or systemic therapies that can cross the blood–brain barrier. Addressing these challenges in a resource-limited context requires tailored, cost-effective strategies that can optimise patient outcomes even in the absence of cutting-edge technologies.

A significant proportion of patients delayed receiving care for more than a year after symptom onset, with 15.5% waiting over 2 years. Delays in presentation and diagnosis are common in low-resource settings, often due to socio-economic barriers, lack of awareness and healthcare access limitations. These delays underscore the absence of a national breast cancer screening program and the inadequacy of existing referral pathways. Early diagnosis and timely initiation of treatment are essential to prevent metastatic spread, but the long delays in seeking care (as seen in the onset–first visit data) highlight the need for community education, better screening programs and improved healthcare access. The high proportion of patients developing metastasis within 6 months of their first visit underlines the importance of aggressive diagnostic work-up at initial presentation. Early-stage treatment and vigilant follow-up may be crucial in preventing or delaying metastatic progression. Interventions targeting both early presentation and timely treatment may help reduce the burden of distant metastases in these patients.

Comparisons with other SSA countries, especially those making progress despite similar challenges, can be instructive. For example, Egypt’s strategic investment in oncology infrastructure and policy-supported screening programs has led to improvements in breast cancer survival [30]. Ghana could adopt a similar phased approach, beginning with regional diagnostic hubs and mobile outreach clinics. As part of aligning with the WHO GBCI, Ghana should prioritise the first of the three GBCI pillars: early detection through awareness and timely diagnosis [34]. Notably, GBCI recommends integrating breast cancer detection into primary care services – a model that can be adapted in Ghana through the CHPS system. Moreover, subsidised breast health services, including clinical breast exams and ultrasound/mammography screening, can be introduced for low-income women as part of the national health insurance scheme.

The findings of the study provide critical data on the clinical and molecular landscape of MBC in Ghana, underscoring the urgent need for tailored interventions that address the unique challenges of MBC management in low-resource settings. The high prevalence of advanced-stage diagnosis, younger age of onset and aggressive molecular subtypes suggest a need for targeted awareness campaigns and early detection programs. Enhancing healthcare infrastructure to provide timely access to diagnostic imaging, biopsy services and pathology support is crucial for improving outcomes. Furthermore, the predominance of palliative care highlights the need for stronger palliative care frameworks, including pain management and psychosocial support, to improve the quality of life in MBC patients [35]. Integrating low-cost, effective treatments such as endocrine therapy, where applicable and developing regional oncology centers could reduce delays and improve outcomes [36]. Implementing telemedicine and mobile health initiatives could also bridge healthcare access gaps. Future research should explore how molecular subtypes and treatment disparities influence these patterns to guide tailored interventions. Urgent policy attention is needed – ranging from the development of national screening guidelines, strengthening of diagnostic infrastructure and expansion of treatment services, to the inclusion of MBC-specific strategies in the revised NCCP. Ghanaian clinicians and researchers must collaborate with the MoH, international partners and civil society to ensure that MBC care evolves to reflect both global best practices and local realities.

## Limitations

The study only included metastases to the following sites (bone, lung, liver and brain). Although the common distant sites for metastasis in breast cancer are bone, liver, lung and brain, other metastasis sites may influence the prognosis of breast cancer patients. The study was conducted at a single institution which may limit the generalisability of the findings regarding the entire sub-Saharan Africa region.

## Conclusion

The study provides critical insights into the socio-demographic, clinical and treatment-related characteristics of MBC in the sub-Saharan African context and demonstrates the metastatic patterns of breast cancer in a resource-limited setting. Bone, lung, liver and brain were the primary metastatic sites, with complex combinations involving these organs reflecting the heterogeneity of the disease. These findings also illustrate disparities between resource-limited and high-resource settings, emphasising the need for context-specific strategies to address the high burden of advanced-stage disease and improve the standard of care for MBC patients. Furthermore, the findings emphasise the need to improve diagnostic capabilities and access to systemic therapy for effectively managing MBC.

## Conflicts of interest

The authors declare no competing interest.

## Funding

This study did not receive any specific funding support from funding agencies in the public, commercial or not-for-profit sectors.

## Data availability

The data used to support the findings of this study are available from the corresponding author upon reasonable request.

## Figures and Tables

**Table 1.** Socio-demographic and clinical characteristics (*N* = 154).

Characteristics	Variables	Frequency (n)	Percentage (%)
Marital status	Single	52	33.8
	Married	76	49.4
	Divorced	9	5.8
	Widowed	17	11.0
Employment status	Employed	94	61.0
	Unemployed	43	27.9
	Retired	17	11.1
Highest level of educational	No formal education	19	12.4
	Primary	43	27.9
	Secondary	69	44.8
	Tertiary	23	14.9
Residence	Urban	106	68.8
	Rural	48	31.2
Menopausal status	Premenopausal	88	57.1
	Postmenopausal	66	42.9
Performance status	ECOG 0	19	12.3
	ECOG 1	24	15.6
	ECOG 2	73	47.4
	ECOG 3	31	20.1
	ECOG 4	7	4.6

ECOG: Eastern Cooperative Oncology Group

**Table 2.** Tumour- and treatment-related characteristics (*N* = 154).

Characteristics	Variables	Frequency (n)	Percentage (%)
Laterality of the affected breast	Left	79	51.3
	Right	71	46.1
	Bilateral	4	2.6
Histological type	Invasive carcinoma, NST	141	91.6
	Invasive lobular carcinoma	4	2.6
	Mucinous carcinoma	4	2.6
	* Other	5	3.2
Histological grade	Grade 1	24	15.6
	Grade 2	58	37.7
	Grade 3	72	46.7
Molecular subtype	Luminal A	34	22.1
	Luminal B (HER-2/Neu+)	20	13.0
	Luminal B (HER-2/Neu-)	27	17.5
	HER-2/Neu-enriched	29	18.8
	Triple negative	44	28.6
Breast surgery	Mastectomy	77	50
	Breast conserving surgery	34	22.1
	Nil	43	27.9
Treatment intent	Curative	0	-
	Palliative	131	85.1
	Best supportive care only	23	14.9
Metastatic setting	De novo metastasis	46	29.9
	Progressive disease	108	70.1
First site of distant metastasis	Bone	59	38.3
	Lung	53	34.4
	Liver	23	14.9
	Brain	19	12.4
Total number of metastatic sites	Solitary	97	63.0
	Double	43	27.9
	Triple	12	7.8
	Quadruple	2	1.3

NST: no special type, * Other histological types comprised metaplastic, adenoid cystic, papillary, cribriform and mixed invasive carcinoma

**Table 3.** Pattern and sequence of occurrence of distant metastasis.

Pattern & sequence of distant metastasis	Frequency (n)	Percentage (%)
Solitary metastasis (*n* = 97)		
°Lung only	32	33.0
°Liver only	16	16.5
°Brain only	11	11.4
°Bone only	38	39.1
Double metastatic sites (*n* = 43)		
°Lung + Liver	7	16.3
°Lung + Brain	1	2.3
°Lung + Bone	8	18.6
°Liver + Lung	2	4.7
°Liver + Bone	2	4.7
°Liver + Brain	1	2.3
°Brain + Lung	1	2.3
°Brain + Liver	1	2.3
°Brain + Bone	3	6.9
°Bone + Lung	11	25.6
°Bone + Liver	2	4.7
°Bone + Brain	4	9.3
Triple metastatic sites (*n* = 12)		
°Lung + Liver + Bone	1	8.3
°Lung + Bone + Liver	1	8.3
°Lung + Brain + Bone	1	8.3
°Bone + Liver + Lung	1	8.3
°Bone + Lung + Liver	1	8.3
°Bone + Lung + Brain	2	16.7
°Liver + Lung + Bone	2	16.7
°Brain + Lung + Bone	2	16.7
°Brain + Liver + Lung	1	8.3
Quadruple metastatic sites (*n* = 2)		
°Lung + Bone + Liver + Brain	2	100

The sites of multiple distant metastasis are listed in order of occurrence
